# Naturally Acquired Antibody Responses to a Synthetic Malaria Antigen AS202.11

**DOI:** 10.1155/2017/6843701

**Published:** 2017-09-12

**Authors:** Rebeka Nazareth, Pius Horumpende, Tolbert Sonda, Arnold Ndaro, Edson Mollel, Eliakim Paul, Emmanuel Athanase, Jaffu Chilongola

**Affiliations:** ^1^Kilimanjaro Christian Medical University College, P.O. Box 2240, Moshi, Tanzania; ^2^Kilimanjaro Clinical Research Institute, P.O. Box 2236, Moshi, Tanzania; ^3^Kilimanjaro Christian Medical Center, P.O. Box 3010, Moshi, Tanzania; ^4^Kibong'oto Infectious Diseases Hospital, P.O. Box 12, Sanya Juu, Siha, Tanzania; ^5^Newala Town Council Hospital, P.O. Box 39, Newala, Tanzania

## Abstract

**Background:**

A major challenge to malaria vaccine development is identification of protective epitopes and respective protective immune responses.

**Objective:**

To characterize naturally acquired Immunoglobulin G (IgG) responses to the synthetic peptide AS202.11, a malaria vaccine candidate.

**Methodology:**

This community based cross-sectional study enrolled 320 participants aged 1 year and above. Demographic information was recorded through interviews. Detection of* P. falciparum* infection was done by microscopy, malaria rapid diagnostic test, and polymerase chain reaction. ELISA was used to detect IgG antibody. Data was analyzed using STATA.

**Results:**

The overall AS202.11 IgG seropositivity was 78.8% (73.9–82.9). Seropositivity by age categories was ≤12 years [74.3% (67.4–80.2)], 13–40 years [85.3% (76.5–91.1)], and >40 years [82.6% (68.7–91.1)]. Compared to the ≤ 12-year-old group, aORs for the other groups were 2.22 (1.14–4.32), *p* = 0.019, and 1.87 (0.81–4.35), *p* = 0.143, for the 13–40-year-old and >40-year-old groups, respectively. The 13–40-year-old group had more seropositive individuals compared to the ≤ 12-year-old group.

**Conclusion:**

We report a high degree of recognition of AS202.11 by IgG elicited by field* P. falciparum *strains, suggesting its close similarity to native* P. falciparum* antigens and possible suitability of the peptide as a future malaria vaccine candidate.

## 1. Introduction

Efforts to control malaria infections are mainly based on mosquito vector control which includes the use of insecticide-treated bed nets and indoor residual spraying, together with treatment using antimalarial medications, mostly artemisinin-based combination therapies (ACTs). Despite all these efforts, malaria continues to be the leading cause of morbidity and mortality. There is a rapid development and spread of insecticide resistance among major malaria vectors in Africa as well as derivatives of artemisinin [1–5] which justifies strengthened efforts to develop alternative novel drugs and vaccines against malaria.

Early efforts of malaria vaccine development focused heavily on the parasite's preerythrocytic stage before it enters human red blood cells but the biggest challenge has been the failure to define specific immune responses associated with protection from malaria. It is well known that symptomatic malaria is caused by blood-stage parasitemia and that acquired protective immunity in humans largely targets blood-stage antigens [6, 7]. This provides a strong justification for developing vaccines based on antigens of blood-stage parasites [6]. Currently, most leading candidate blood-stage antigens are merozoite proteins, located on the merozoite surface or within the apical organelles and more than 30* P. falciparum* malaria vaccine candidates are at either advanced preclinical or clinical stages of evaluation [8–11]; however only the RTS, S/AS01, a preerythrocytic stage hybrid recombinant protein vaccine, has completed Phase 3 evaluation. One of the major impediments is the difficulty in the identification of protective epitopes and understanding the nature and targets of protective immune responses [6]. It is reasonable therefore that more research should be directed to developing blood-stage vaccines.

In the search for epitopes that could induce protective immunity against malaria, scientists have developed many synthetic peptides that are closely related to parasite antigens for development of future malaria vaccines. Alpha (*α*) helical coiled motifs in malaria antigens, such as MSP3 and MSP6, are important oligomerization subunits and targets of malaria protective antibodies [12]. When *α*-helical coiled motifs are separated from the whole protein, they readily fold into the same stable oligomeric structure [13]. The synthetic peptide AS202.11 (qleektkqyndlqnnmktikeqnehlknkfqsmgk), described in detail elsewhere [14, 15], is an *α*-helical coiled motif. Previous studies on antibody responses to this peptide showed a modest association with reduced risk of clinical malaria in children resident in the Kilifi district of Kenya and West Africa [9, 11, 16]. The current study evaluated the extent to which naturally acquired IgG antibody responses to* P. falciparum* recognize the synthetic peptide AS202.11 in a Tanzanian high malaria transmission site, in terms of seropositivity across different age groups of individuals in the study community.

## 2. Methodology

### 2.1. Study Design, Study Sites, and Population

The current study was a community based cross-sectional study. The study was conducted in Bondo and Kwamgwe villages in Tanga region of North Eastern Tanzania, which is situated about 300 km from Dar es Salaam along the Dar es Salaam-Arusha highway from March to December 2016. The study site has a stable and perennial malaria transmission, although the most cases of* P. falciparum* infections occur after the long rains (March–June) and the short rains (October-November) of the year. It is 7 km off the Moshi-Dar es Salaam highway. The study area has two rainy seasons per year, which denotes the peaks of malaria transmission. The prevalence of malaria was 20.5% in 2016 [17] and an approximate entomological inoculation rate (EIR) of 100 infectious bites per person per year (unpublished data). The area is located at 309 meters above sea level, 5°22′60′′N and 38°34′60′′E, with a population of 7970 according to 2012 Tanzania census, most of whom are peasants (National Bureau of Statistics, 2013). This study was nested within a larger study that was designed to evaluate the protective roles and changing dynamics of subclasses of IgG antibodies to* P. falciparum* in relation to incidences of clinical malaria under the Building Stronger University Program (Phase 2) and Medical Education Partnership Initiative (MEPI). The study enrolled 320 participants aged 1 year and above, who were residents of the study areas. The study sites were chosen conveniently, while participants were selected randomly from 100 randomly selected households. Members from those selected households were asked to go to the ward research-health facility for sample collection. A short questionnaire was used to obtain demographic information from participants.

### 2.2. Laboratory Procedures and Assays

Thick and thin blood smears for malarial microscopy were prepared as described elsewhere [17]. Briefly, a blood sample of 0.5–1 mL was taken by venipuncture from all consenting participants and about 10 ul of whole blood was subjected to rapid diagnostic test for malaria diagnosis (SD BIOLINE Malaria Ag P. f/Pan). Children under the age of 5 who were found to be malaria positive by rapid diagnostic test were immediately treated with antimalarial according to national and WHO guidelines. Remaining whole blood was centrifuged at 700 ×g for 5 min to obtain plasma which was stored at −20°C till when serological (enzyme linked immunosorbent assay) test was performed. Adults with fever and positive by MRDT were treated with Artemether-Lumefantrine (ALu), the first-line antimalarial drug in Tanzania.

### 2.3. Enzyme Linked Immunosorbent Assay (ELISA)

Immunoglobulin G (IgG) to AS202.11 was detected by performing an indirect enzyme linked immunosorbent assay (ELISA) based on a protocol developed by Afro Immunoassay Consortium. Briefly, 96-well flat-bottom microtitre plates (MaxisorpNunc, Roskilde, Denmark) were coated overnight at 4°C with purified his-tag produced recombinant AS202.11 antigen (0.5 g/ml) diluted in phosphate buffered saline (PBS). The plates were blocked with 3% powdered skimmed milk-containing-phosphate buffered saline at pH of 7.4 and 0.1% Tween-20 for one hour. Plasma from test samples was diluted to 1 : 200 whereas positive and negative control samples (primary antibodies) were diluted to 1 : 50 in dilution buffer (PBS with 1% powdered-milk and 0.1% Tween-20). One hundred microliters was added to plates in duplicate. The plates were then incubated at room temperature for one hour on a rocker platform, and then 100 uL of the peroxidase-conjugated goat anti-human IgG, diluted to 1 : 8000, was added in respective wells and incubated at room temperature for one hour in the dark place for 30 minutes. Plates were washed four times with washing buffer (PBS with 0.1% Tween-20 and 0.5 M NaCl) between steps above and six times after incubation with the peroxidase-conjugated goat anti-human antibodies. The bound secondary antibodies were quantified by staining with 50 *μ*l of ready to use TMB (3, 3′, 5, 5′-tetramethylbenzidine) and incubated at room temperature for 20 minutes in the dark. The reaction was stopped by addition of 50 *μ*l of 0.2 M H2SO4 into each well, and then plates were read at 450 nm using ELISA plate reader (BioRad, Chi, USA) using Gene5 software. Cut-off optical density (OD) values were calculated based on the negative controls in each plate. The test sample was determined as positive if its optical density (OD) value was greater than the mean OD of negative control +2SD and when the OD value was less than the mean OD of negative controls +2SD the test sample was regarded as negative.

### 2.4. Data Processing and Statistical Analysis

A well-designed data abstraction form was used for data collection. Data collected included demographic, clinical, and immunological (IgG to AS202.11). Data were entered into Microsoft Excel 2007. Stata 14 (College Station, Texas 77845, USA) was used for data analysis. The age of participants was categorized into three groups of ≤12, 13–40, and >40 years. Chi square test was used to test the differences in proportions and determine relationships between categorical variables. Correlation coefficient between anti-AS202.11 IgG concentrations (OD) and age was determined. Logistic regression was used to identify factors that independently associate with AS202.11 IgG seropositivity. A two-tailed *p* value of 0.05 or less was considered statistically significant.

## 3. Results

A total of 320 participants were included in the analysis of which 210 (65.6%) were females. One hundred seventy-nine (55.9%) were aged 12 years and below. Out of 320, 252 (78.8%) of the participants were seropositive for IgG antibodies to the AS202.11 peptide. One hundred and one (96.2%) of malaria infection was due to* P. falciparum*. Of the 320 individuals tested for malaria infection, fifty-seven (17.8%) were positive for malaria by microscopy, whereas 155 (48.4%) were malaria positive by MRDT (Table 1).

**Figure 2:**
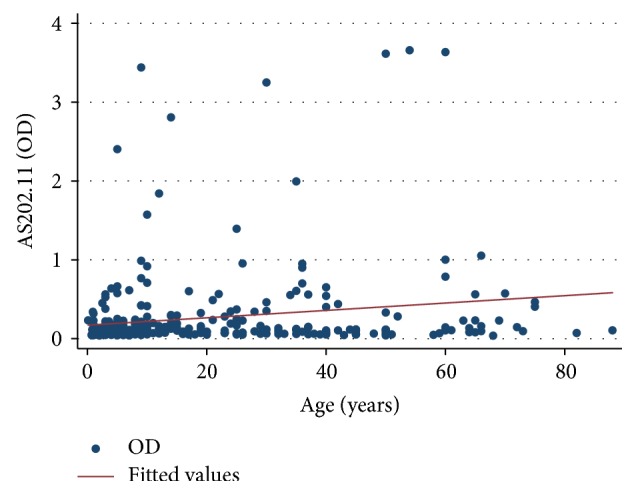
Scatter plot showing the relationship between anti-AS202.11 IgG concentrations (OD) and age of participants.

Analysis to determine correlation between IgG concentration and AS202.11 IgG seropositivity was performed and results are presented in Figure 2. Concentration (optical density) of IgG to AS202.11 showed a positive correlation with AS202.11 IgG seropositivity with a correlation coefficient (*r*^2^) of =0.031. Bivariate linear regression analysis showed that every one-year increase in age corresponds to an increase in AS202.11 IgG seropositivity by 0.0047 (95% CI: 0.0020–0.0080), *p* < 0.001.

AS202.11 IgG seropositivity in the different age categories of study participants was determined (Table 2). The overall AS202.11 IgG seropositivity (95% CI) was 78.8% (73.9–82.9). The seropositivity by age categories were ≤12 years: 74.3% (67.4–80.2), 13–40 years: 85.3% (76.5–91.1), and >40 years: 82.6% (68.7–91.1).

Regression analysis to determine and quantify the association between IgG seropositivity and AS202.11 with other variables was performed (Table 3). The youngest group of ≤12 years was used as the reference group in the analyses. Unadjusted OR (95% CI) for AS202.11 IgG seropositivity among participants aged 13–40 years was 2.0 (1.04–3.87), *p* = 0.039, compared to those aged ≤12 years while the OR (95% CI) among those aged >40 was 1.64 (0.71–3.78), *p* = 0.243, compared to those aged ≤12 years. Adjusted OR (95% CI) among participants aged 13–40 years was 2.22 (1.14–4.32), *p* = 0.019, whereas the OR (95% CI) among those aged >40 was 1.87 (0.81–4.35), *p* = 0.143. Unadjusted OR (95% CI) for AS202.11 IgG seropositivity among participants with malaria positive by microscopy was 2.62 (1.07–6.40), *p* = 0.034, compared to those with malaria negative by microscopy. Adjusted OR (95% CI) among participants with malaria positive by microscopy was 3.03 (1.23, 7.49), *p* = 0.016, compared to those with malaria negative by microscopy. Body temperature, MRDT, and PCR had insufficient observations to perform a multivariate logistic regression although on a univariate regression they were statistically significant predictors of AS202.11 IgG seropositivity. There was no statistical difference in the seropositivity in the ≤12-year-old and the >40-year-old group nor the 13–40-year-old and the >40-year-old group. However, the 13–40-year-old group had a statistically higher proportion of seropositive individuals compared to the ≤12-year-old group.

## 4. Discussion

In the present study, about three-quarters (78.8%) of participants were seropositive for total IgG antibodies to the AS202.11 peptide. Analyses for statistical differences in terms of seropositivity show statistical differences between the youngest (≤12 years) and the other age categories; thus the youngest category had a significantly lower seropositive proportion compared to the older groups and the overall seropositivity. The middle age (13–40 years) and the older (>40 years) categories did not differ significantly with the overall seroprevalence. This may explain the age-dependent development of malaria immunity considered to be an accumulation of immune responses against poorly immunogenic conserved determinants [17].

Previous studies had reported similar findings on the age-dependent protective roles of IgG antibodies to several* P. falciparum* asexual blood-stage antigens [16–20], which may most likely represent the additive effect of persistent exposure to the many parasite antigens circulating in the local population. The trend also may imply slow maturation of protective immune responses against malaria [13]. Our observation of about four-fifth of the local community to respond to the AS202.11 peptide and the increased peptide specific antibody levels in older individuals for the AS202.11 peptide suggests the peptide conformations are closely similar to those of native parasite proteins. This was expected since the AS202.11 peptide contains predicted-helical coiled-coil domains which fold into the same stable oligomeric structure as isolated native protein fragments [14, 16]. It also suggests the ability of the synthetic peptide to elicit IgG responses in a similar manner to the native parasite proteins.

We show in this study also that individuals who were positive for malaria were more seropositive for the AS202.11 peptide than those who were not infected. This is similar to what was previously reported by Agak and colleagues in a study done in Kenya [16]. As most of the infections were due to* P. falciparum, *seropositivity to the AS202.11 peptide suggests close similarity of the peptide to particularly* P. falciparum* antigens. Development of malaria vaccines has traditionally been focused on a variety of candidate surface antigens from the different stages of the malaria parasite [6, 16, 17, 19–21]. Unfortunately, to date immune responses associated with protection against malaria have not been precisely defined. In the current study, we determined if naturally acquired IgG antibody can recognize the synthetic malaria peptide AS202.11 and the extent of the recognition in the form of seroprevalence as way of extending our range for possible future vaccine candidate molecules. Our study had the inherent limitation of being a cross-sectional study, with a small sample size for the analyses which could have affected statistical power of associations. However, our findings are valuable and provide important clues regarding IgG antibody responses against a synthetic peptide as a malaria vaccine candidate.

## 5. Conclusion

Our findings show a high degree of recognition of the AS202.11 by IgG elicited by field* P. falciparum* strains. Specifically, our findings have elucidated the recognition of the synthetic peptide AS202.11 by natural IgG and correlations between anti-AS202.11 IgG antibody concentrations (ODs) and seropositivity across the age of participants. These are preliminary and basic attributes of a vaccine component. This infers the possibility of further characterization of the AS202.11 peptide and types and protective roles of immune responses to the peptide as a promising malaria vaccine candidate.

## Figures and Tables

**Figure 1:**
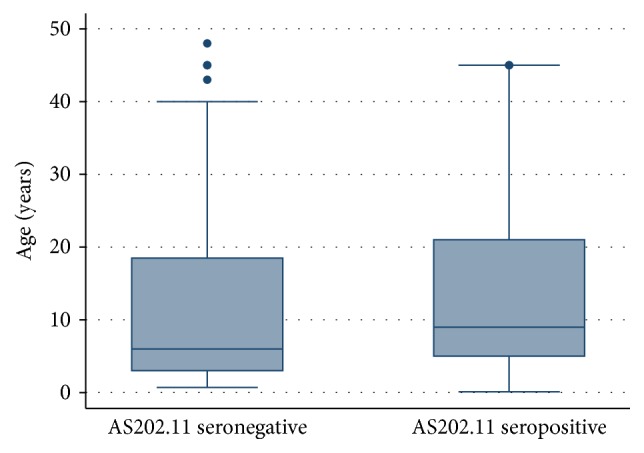
The relationship between AS202.11 seropositivity and age.

**Table 1:** Description of study population and frequencies of measurements (*n* = 320).

Variable	Frequency
*Age (years)*	No (%)
≤12	179 (55.9)
13–40	95 (29.7)
>40	46 (14.4)
^a^Parasitemia, median (IQR)^@^	5.15 (2.64–6.19)
AS202.11 OD^#^, median (IQR)	0.10 (0.07–0.20)
*Body temp*	
≤37.5°c	191 (69.2)
>37.5°c	85 (30.8)
*Microscopy*	
Negative	263 (82.2)
Positive	57 (17.8)
*mRDT* ^b^	
Negative	165 (51.6)
Positive	155 (48.4)
*PCR* ^c^	
Negative	200 (62.5)
Positive	120 (37.5)
*Seropositivity*	
Non Responders	68 (21.2)
Responders	252 (78.8)
*Sex*	
Female	210 (65.6)
Male	110 (34.4)
*Speciation*	
Non-*Plasmodium falciparum*	4 (3.8)
*Plasmodium falciparum*	101 (96.2)

*Key*. ^a^Parasitemia is in logarithmic form (log_10_). ^@^Interquartile range. ^b^Malaria rapid diagnostic test. ^c^Polymerase chain reaction. ^#^Optical density. AS202.11 IgG responders had a relatively higher median age than the median age of nonresponders (Figure 1), although nonresponder group had extremes of a few high age values (outliers).

**Table 2:** AS202.11 seroprevalence in each of the age categories.

Age category (years)	AS2022.11 seroprevalence (%)	(95% CI)
Overall	78.8%	73.9–82.9
≤12	74.3%	67.4–80.2
13–40	85.3%	76.5–91.1
>40	82.6%	68.7–91.1

**Table 3:** Regression analysis of association between seropositivity and AS202.11 with other parameters.

Variable	Univariate			Multivariate		
	OR	(95% CI)	*p* value	OR	(95% CI)	*p* value
*Age (year)*						
≤12	1.00					
13–40	2.00	(1.04–3.87)	0.039	2.22	(1.14–4.32)	0.019
>40	1.64	(0.71–3.78)	0.243	1.87	(0.81–4.35)	0.143
*Body temp*						
<36.5°c	1.00					
≥36.5°c	0.56	(0.31–1.03)	0.064	—		
*Gender*						
Female	1.00					
Male	0.88	(0.50–1.53)	0.640	—		
*MRDT*						
Negative	1.00					
Positive	2.34	(1.33–4.11)	0.003	—		
*PCR*						
Negative	1.00					
Positive	1.72	(0.96–3.10)	0.068	—		
*Microscopy*						
Negative	1.00					
Positive	2.62	(1.07–6.40)	0.034	3.03	(1.23, 7.49)	0.016
